# Oxygen Availability on the Application of Antimicrobial Photodynamic Therapy against Multi-Species Biofilms

**DOI:** 10.3390/pathogens12070904

**Published:** 2023-07-03

**Authors:** Min Nie, Jingmei Yang, Alessandra Nara de Souza Rastelli, Yuqin Shen, Dongmei Deng

**Affiliations:** 1 Department of Periodontics, Affiliated Stomatology Hospital of Guangzhou Medical University, Guangdong Engineering Research Center of Oral Restoration and Reconstruction, Guangzhou Key Laboratory of Basic and Applied Research of Oral Regenerative Medicine, Guangzhou 510182, China; minnie@gzhmu.edu.cn; 2 State Key Laboratory of Oral Disease & National Clinical Research Center for Oral Diseases, Department of Periodontics, West China Hospital of Stomatology, Sichuan University, Chengdu 610041, China; yangjm@scu.edu.cn; 3 Department of Preventive Dentistry, Academic Centre for Dentistry Amsterdam, University of Amsterdam and Vrije Universiteit Amsterdam, 1081 LA Amsterdam, The Netherlands; d.deng@acta.nl; 4 Department of Restorative Dentistry, School of Dentistry, Sao Paulo State University-UNESP, Araraquara 14801–903, Brazil; alessandra.nara-souza-rastelli@unesp.br

**Keywords:** biofilm, curcumin, methylene blue, photodynamic therapy, oxygen

## Abstract

Methylene blue (MB)- or Curcumin (Cur)-based photodynamic therapy (PDT) has been used as an adjunctive treatment for periodontitis. Its actual clinical efficacy is still in question because the lack of oxygen in a deep periodontal pocket might reduce the PDT efficacy. We aim to investigate the effect of oxygen on PDT efficacy and to examine if the addition of hydrogen peroxide (HP) could improve PDT performance anaerobically. To this end, we cultured 48 h saliva-derived multi-species biofilms and treated the biofilms with 25 µM MB or 40 µM Cur, HP (0.001%, 0.01% and 0.1%), light (L-450 nm or L-660 nm), or combinations thereof under ambient air or strictly anaerobic conditions. MB- and Cur-PDTs significantly reduced biofilm viability in air but not under anaerobic conditions. HP at 0.1% significantly enhanced the killing efficacies of both MB- and Cur-PDTs anaerobically. The killing efficacy of Cur-PDT combined with 0.1% HP was higher anaerobically than in air. However, this was not the case for MB-PDT combined with 0.1% HP. In conclusion, this study demonstrated that the biofilm killing efficacies of MB- and Cur-PDTs diminished when there was no oxygen. HP at 0.1% can enhance the efficacy of PDT performed anaerobically, but the level of enhancement is photosensitizer-dependent.

## 1. Introduction

Periodontitis is an inflammatory disease associated with dental plaque. At the advanced stage, it can lead to tooth loss and reduce the quality of life. It was reported that periodontal disease affected 1.1 billion persons globally in 2019 [1]. Scaling and root planing (SRP) is the cornerstone of nonsurgical periodontal therapy [2], but its efficacy is limited in sites with impaired access, such as furcation areas, deep periodontal pockets, or residual bacterial deposits. Adjunct treatments to SRP are usually necessary for these clinical cases. 

Photodynamic therapy (PDT) has been recommended to be an adjunct treatment to SRP [3]. It is based on the local application of a photosensitizer (PS), which can absorb light of the appropriate wavelength (visible or near-infrared) in the presence of molecular oxygen, leading to the production of antimicrobial or cytotoxic reactive oxygen species [4]. Ample in vitro studies have illustrated that PDT can significantly reduce the viability of multi-species biofilms which contain both aerobic and anaerobic bacteria [5,6,7,8]. Data from clinical studies also indicated that patients with chronic periodontitis showed significantly improved clinical parameters; for example, reduced probing depth and/or bleeding score, after being treated with SRP followed by PDT, compared to those treated with SRP alone [9,10]. However, the adjunct efficacy of PDT was less promising in patients with aggressive periodontitis or severe cases of chronic periodontitis [11]. These clinical cases are usually featured with deep periodontal pockets of ≥7 mm [12].

Oxygen availability is a critical factor for the optimal application of PDT in periodontal treatment. The working mechanism of PDT shows that this therapy is oxygen-dependent [13]. PDT has reportedly failed to confer cytotoxicity in hypoxic tumors [14]. In periodontitis patients, their periodontal pocket is considered to be hypoxic. Clinical measurements have shown that the oxygen levels in an untreated periodontal pocket could be as low as 0.7%. Oxygen tension increased significantly with increasing pocket depth [15,16]. The low oxygen level in the deep pocket was believed to determine the composition of subgingival biofilms, where strict anaerobes were more prevalent in the patients with deeper periodontal pockets [17]. These strict anaerobes, for example, the known red complex species, have been associated with the severeness of periodontal diseases [18]. Therefore, the low efficacy of PDT in treating patients with severe periodontitis might be related to the availability of oxygen at the local site. 

Methylene blue- or Curcumin-based PDTs have been used as adjunctive treatments for periodontitis. Methylene blue (MB), a phenothiazinium dye, carries a positive charge with a single oxygen quantum yield below 0.5. It generates reactive oxygen species (ROS), mainly via the type I mechanism, and shows strong absorption in the red spectrum (600–680 nm) [19]. It has received clinical approval [20] for use in humans in combination with light. Therefore, it is a popular photosensitizer in clinical studies. Unlike MB, Curcumin (Cur) acts nearly quantitatively according to the type I mechanism and displays absorption in the UV/blue spectrum (300–500 nm). The photokilling effect occurs at relatively short drug–light intervals [19]. Until now, the antimicrobial efficacy of Cur-based PDT has been demonstrated primarily in in vitro studies, but seldom in clinical settings. Only a few studies have been focused on the antimicrobial efficacy of Cur-based PDT [21,22]. In a clinical study with a split-mouth design, Sreedhar et al. [23] showed that Cur-PDT significantly reduced the amount of certain bacterial species compared to the SRP or Cur alone groups, but did not improve any clinical parameters, such as pocket depth and attachment loss. Since Cur is a natural plant extract, it is recognized as a product of high safety and low toxicity. This is supported by the United States Food and Drug Administration (FDA), which claims it as a compound “generally recognized as safe” [24]. Therefore, the application of Cur-PDT in treating periodontitis is worth further investigation, despite the uncertain clinical efficacy. 

The aim of this study is two-fold: (1) to explore the effect of oxygen on the antimicrobial efficacies of MB- and Cur-PDT; (2) to examine if an additional hydrogen peroxide (HP) treatment could improve the treatment efficacy of PDTs, in particular when there is a lack of oxygen. To this end, the PDT antimicrobial efficacy was evaluated in an in vitro multi-species biofilm model, which can better resemble the multi-species clinical infections than the single-species biofilm model. 

## 2. Materials and Methods

### 2.1. Multi-Species Biofilm Formation

The multi-species biofilms were grown in an Amsterdam Active Attachment (AAA) model using human saliva as an inoculum [25]. The AAA model consists of a stainless-steel lid and a 24-well tissue culture plate (Greiner Bio-One, Alphen a/d. Rijn, The Netherlands). A total of 24 clamps were fixed on the lid. Each clamp holds one round glass coverslip with a diameter of 10 mm (Menzel, Braunschweig, Germany), which is used as the substratum for biofilm formation.

The saliva inoculum was collected and pooled from 10 systemically and orally healthy adult donors. Prior to saliva donation, the donors were asked not to brush their teeth for 24 h and abstained from food and drink intake for 2 h. The unstimulated saliva pool was mixed with 60% glycerol at 1:1 ratio and stored at −80 °C till further application. This protocol was approved by the Medical Ethical Committee of the VU University Medical Center Amsterdam (document number 2011/236).

To form a biofilm, the pooled saliva was diluted with biofilm growth medium (BGM) containing 10% fetal calf serum (FCS) at a ratio of 1:50 and then added to a sterile 24-well plate (1.3 mL/well). The plate was covered with the lid containing glass coverslips and incubated anaerobically (10% CO_2_, 10% H_2_ and 80% N_2_) at 37 °C. The BGM was refreshed after 8 h the first time and then once every 20 h. The 48 h biofilms were subjected to PDT and/or HP treatments.

The BGM contains 2.5 g/L mucin (Sigma, Saint Louis, MO, USA), 2.0 g/L Bacto peptone (Difco, Detroit, MI, USA), 2.0 g/L trypticase peptone (BBL, Cockeysville, MD, USA), 1.0 g/L yeast extract (BD Diagnostic Systems, Sparks, MD, USA), 0.35 g/L NaCl, 0.2 g/L KCl, 0.2 g/L CaCl_2_, 1 mg/L hemin (Sigma) and 2 mg/L vitamin K1 [26], with 50 mM PIPES at pH 7.0.

### 2.2. PDT and HP Treatments

MB, Cur and HP (30%) were purchased by Sigma Aldrich (Sigma Aldrich, Saint Louis, MO, USA). MB stock solution (20 mM) was prepared in distilled water, whereas Cur stock solution (20 mM) was prepared in 100% Dimethyl sulfoxide DMSO (Sigma Aldrich, Saint Louis, MO, USA). Before use, both stock solutions were filtered through a 0.20 µm membrane filter (Sarstedt, Hildesheim, Germany). The working solutions of both MB and Cur were diluted in sterile distilled water. Both working solutions were kept in the dark by wrapping the tubes with tinfoil. The HP test concentrations were 0.001%, 0.01% and 0.1%, which were diluted from the 30% stock solution with distilled water.

The 48 h multi-species biofilms were rinsed with distilled water to remove loosely attached bacterial cells and then subjected to the following treatments: 1. distilled water (served as a no-treatment control); 2. 25 µM MB or 40 µM Cur alone (for checking the dark toxicity of MB or Cur); 3. light alone (L450-wavelength of 450 nm or L660-wavelength of 660 nm, (for checking if the light itself could cause biofilm viability reduction); 4. different concentrations of HP alone (for checking the antimicrobial efficacy of HP); 5. PS + light (25 µM MB L660 or 40 µM Cur + L450, (to demonstrate the killing efficacy of MB-PDT and Cur-PDT without addition of HP); 6. PS + light + HP (to demonstrate the killing efficacy of MB-PDT and Cur-PDT with HP). The water or PS or PS + HP solutions were added directly to a 24-well plate (1.3 mL/well). The biofilms were incubated in the dark at room temperature for 5 min and then irradiated by a light-emitting diode (LED) light using a BioTable (MMOptics, Sao Carlos, Sao Paulo, Brazil). The final energy intensity was 15 J/cm^2^ for both MB and Cur. To achieve this energy intensity, the irradiation durations were 13.15 min for MB (L660) and 11.37 min for Cur (L450) because the irradiation power density was 19 mW/cm^2^ for L660 and 22 mW/cm^2^ for L450, according to the factory settings of BioTable. 

All treatments were performed in ambient air or under strictly anaerobic conditions in an anaerobic chamber. The experiment was repeated 3 times, with triplicate samples in each treatment group per experiment.

### 2.3. Viable Cell Counts 

A plate-counting method assessed the viability of the multi-species biofilms after various treatments. After treatments, each coverslip with biofilms was rinsed with sterile distilled water before placing in a tube containing 2 mL of cysteine peptone water (CPW, 5 g yeast extract, 1 g peptone, 8.5 g NaCl, 0.5 g L-cysteine HCl per liter, adjusted to pH 7.2). The biofilm cells were removed from the coverslip by vortexing for 30 s, followed by sonication on ice for 1 min with a pulse of 1 s at an amplitude of 40% (Vibra CellTM, Sonics and Materials Inc., Newtown, CT, USA). The samples were serially diluted and plated onto tryptic soy agar containing 5% sheep blood, 5 mg/mL of hemin and 1 mg/mL of menadione. The plates were incubated anaerobically at 37 °C for 7 days before counting colony-forming units (CFUs). 

### 2.4. Statistical Analysis

Statistical analyses were carried out using the Statistical Package for Social Science (SPSS 22.0, IBM Corporation, Armonk, NY, USA). All CFU values were log-transformed before analysis. The overall effects of various HP concentrations and the PDT (light alone, PS alone and light + PS) on biofilm viability were analyzed by two-way ANOVA. The effects of PDT conditions on biofilm viability at each HP concentration was further analyzed by one-way ANOVA, followed by the post-hoc test Dunnett T3. The effect of MB-PDT or Cur-PDT on biofilm viability without HP or in the presence of 0.1% HP was analyzed by one-way ANOVA, followed by the post-hoc test Dunnett T3 (heterogeneous variance) or Student–Newman–Keuls (homogeneous variance). The effects of the PDTs performed in air and under anaerobic conditions were analyzed separately. Differences were considered statistically significant at *p* < 0.05.

## 3. Results

The multi-species biofilm layer was visible on the surfaces of all coverslips after 48 h. These biofilms were treated on a laboratory bench under ambient air or in an anaerobic chamber. Since Cur is not water soluble, it was first dissolved in 100% DMSO and then diluted 500 times with distilled water to reach the working concentration of 40 µM. Therefore, the Cur treatment solution contains 0.2% DMSO. The influence of 0.2% DMSO on biofilm viability was examined in a pilot experiment. The viability was unaffected after incubating the biofilm in the biofilm medium containing 0.2% DMSO for 24 h. In the current study, distilled water, the solvent used to dissolve MB, was used as the control treatment.

### 3.1. The Killing Efficacies of MB-PDT and Cur-PDT Performed in Air or Anaerobically 

**Figure 1:**
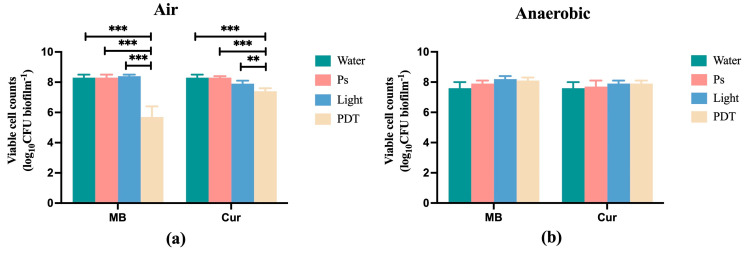
The killing efficacies of MB-PDT and Cur-PDT under air (**a**) and anaerobic (**b**) conditions. The 48 h multi-species biofilms were subjected to various treatments and the biofilm viable cell counts were examined by agar plate counting method. MB: methylene blue; Cur: curcumin; Water: distilled water; PS: 25 µM MB or 40 µM Cur; Light: wavelength of 450 nm (Cur) or wavelength of 660 nm (MB) alone; PDT: PS (Cur or MB) combined with light (450 nm or 660 nm). ** refers to statistical significance with *p* < 0.01, *** refers to statistical significance with *p* < 0.001.

Figure 1a shows that when the treatments were performed in air, the biofilm viable cell counts were significantly reduced from logCFU of 8.3 ± 0.2 in the water group to 5.7 ± 0.7 in MB-PDT and 7.4 ± 0.2 in Cur-PDT groups. However, when the treatments were performed anaerobically (Figure 1b), neither MB-PDT nor Cur-PDT affected biofilm viability. The PS or light-alone treatments did not affect the biofilm viability, irrespective of oxygen availability.

### 3.2. The Killing Efficacies of MB-PDT and Cur-PDT in the Presence of HP

**Figure 2:**
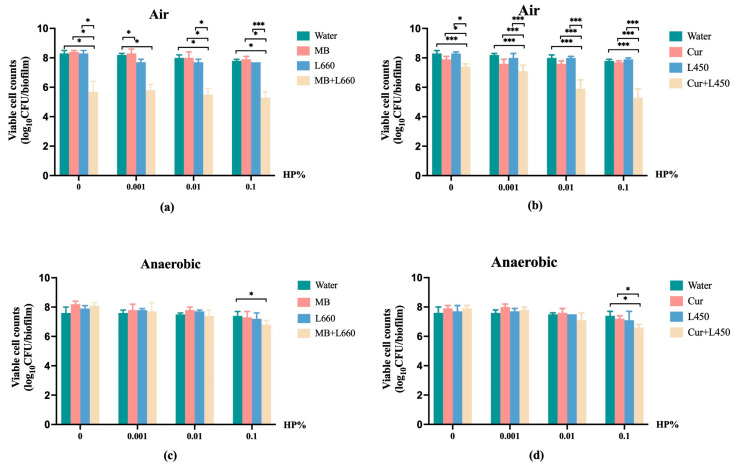
The killing efficacies of MB-PDT (**a**,**c**) and Cur-PDT (**b**,**d**) in the presence of different concentrations of HP (0.001%, 0.01% and 0.1%) under air or anaerobic conditions. The CFUs were calculated to illustrate the additional effects of HP. HP: hydrogen peroxide; Water: distilled water; MB: 25 µM methylene blue; Cur: 40 µM curcumin; L660: wavelength of 660 nm light; L450: wavelength of 450 nm light. * refers to statistical significance with *p* < 0.05; *** refers to statistical significance with *p* < 0.001.

Next, we treated the multi-species biofilms with various concentrations of HP in combination with PDT. We hypothesize that the HP treatment might improve the PDT killing efficacy by elevating oxygen concentration locally under anaerobic conditions [27]. Figure 2 demonstrates an overview of the killing efficacies of the PDT treatments (water, PS alone, light alone and PDT) under each HP concentration. The biofilm viability in each PDT treatment set shows a similar pattern irrespective of HP concentrations. The two-way ANOVA analysis shows that the overall killing efficacy of PDT treatments was not significantly enhanced by the additional of HP. However, it seems that the addition of 0.1% HP increased the killing efficacy of MB-PDT and Cur-PDT. The effects of this HP concentration were therefore selected to analyze further. We also analyzed the killing efficacy of HP alone in the water group. HP alone led to a dose-response reduction in the biofilm viability. This dose-response reduction was marginally significant when the treatments were conducted in air (*p* = 0.07), but not significant when they were conducted anaerobically.

### 3.3. The Killing Efficacies of MB-PDT and Cur-PDT Combined with 0.1% HP Performed in Air or Anaerobically

**Figure 3:**
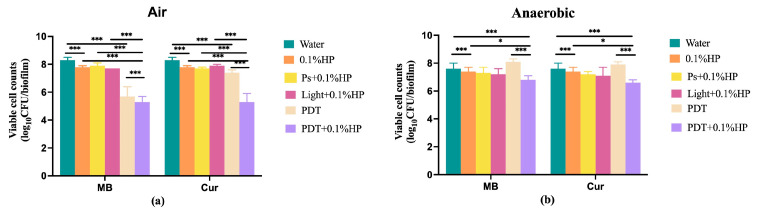
Viable cell counts of 48 h multi-species biofilms after various treatments performed under air (**a**) or anaerobic (**b**) conditions. HP: Hydrogen peroxide; PS: 25 µM methylene blue (MB) or 40 µM curcumin (Cur); Light: wavelength of 450 nm (for Cur) or wavelength of 660 nm (for MB); PDT: PS + light.; Water: distilled water. * refers to statistical significance with *p* < 0.05; *** refers to statistical significance with *p* < 0.001.

We further analyzed the killing efficacies of MB- and Cur-PDTs in the presence of 0.1% HP (Figure 3). When the treatments were performed in the air (Figure 3a), 0.1% HP alone significantly reduced biofilm viability, as compared to the water group. When PS alone or light alone was combined with 0.1% HP, the killing efficacy was similar to HP alone. The additional 0.1% HP did not enhance the killing efficacy of MB-PDT but significantly enhanced that of Cur-PDT. When the treatments were performed anaerobically (Figure 3b), 0.1% HP alone, 0.1% HP combined with PS alone, or light alone did not significantly reduce biofilm viability, as compared to the water group. But additional 0.1% HP clearly enhanced the killing efficacy of both MB-PDT and Cur-PDT. The biofilm viability in the HP + PDT groups was significantly lower than the HP alone or PDT alone groups. Furthermore, the biofilm viability reductions caused by the combined treatments were much less under anaerobic conditions than under ambient air.

## 4. Discussion

PDT has been considered a promising adjunct treatment to SRP for periodontitis [3]. However, its efficacy in patients with aggressive periodontitis or severe cases of chronic periodontitis is still questioned [11]. One of the obstacles to a successful PDT could be its requirement for oxygen. Since inflammatory periodontal pockets are known to be hypoxic, the efficacy of local PDT might be greatly reduced. In this in vitro study, we investigated the killing efficacy of PDT against multi-species biofilms in an environment where oxygen levels were well-controlled. Our data revealed that the biofilm killing effects of MB-PDT and Cur-PDT were indeed undetectable under strictly anaerobic conditions, even though they were clearly visible when the treatments were conducted in air. 

Moreover, we demonstrated that HP at 0.1% could significantly enhance the killing efficacies of PDT. However, the level of enhancement was PS-dependent: the addition of 0.1% HP enhanced the killing efficacy of Cur-PDT under ambient air and anaerobic conditions. It also enhanced the killing efficacy of MB-PDT under anaerobic conditions but did not change the efficacy of MB-PDT under ambient air.

It is known that a sufficient oxygen level is critical for an optimal photodynamic action [28]. Previously, Huang et al. [29] have shown that the killing of *Staphylococcus aureus* and *Escherichia coli* suspensions by MB-PDT was completely abrogated when oxygen was removed by bubbling with nitrogen. The results of the current study not only confirmed the findings of Huang et al. [29] in a multi-species biofilm model, but also showed that the killing effect of Cur-PDT is also oxygen-dependent. Other PSs, such as fullerene [30] and rose bengal [31], have also been reported to have oxygen-dependent antibacterial efficacy. In contrast, Hope et al. [32] found that the 405 nm light source achieved more than 90% of the killing of *Porphyromonas gingivalis* suspension cells under anaerobic conditions due to the endogenous PS, porphyrins. The authors proposed that the type I mechanism, distinct from the type II mechanism, which was less oxygen dependent, might cause this lethal photosensitization. However, this hypothesis cannot explain why the function of MB and fullerene was oxygen-dependent, since both PSs are known to act by the type I mechanism [8,30]. Therefore, the type I or II mechanism alone might not be sufficient to explain the oxygen-independent photokilling shown by Hope et al. Nevertheless, sodium azide induced a strong oxygen-independent bacterial killing when applied with MB-PDT or fullerene-PDT [28]. Hence, the azide anion was proposed as an additional electron donor that can potentiate PDT in the presence and absence of oxygen. 

Unfortunately, the azide anion is very toxic to mammalian cells, making it unsuitable for clinical infection treatment. Therefore, we investigated if HP is able to restore the killing effects of PDT under anaerobic conditions. HP is a common antimicrobial used in dental clinics [33]. The FDA has approved using HP at less than 3% as an oral debriding agent. It has been shown that the addition of 0.3% HP enhanced the antimicrobial effects of MB-PDT [34,35,36], although another study [37] did not find any enhancement at 40 µM (0.12%) HP. Our data showed that the additional 0.1% HP could enhance the killing efficacy of MB-PDT under anaerobic conditions, but it could not restore its efficacy to the level when the treatment was performed with air. The mechanisms for the enhancement of PDT efficacy by HP are not yet clear. Several hypotheses have been put forward. HP might elevate oxygen concentration locally or act as an electron receptor to produce reactive oxygen species [27,35]. It is also possible that the uptake of a PS was improved after exposing the microbes to HP. Nevertheless, since HP is already in use in the dental clinic, combining HP with PDT may be useful in treating periodontitis patients with deep periodontal pockets. Clinical studies are needed to verify the in vivo antimicrobial efficacy of the combined treatment of HP and PDT. Furthermore, potassium iodide has been suggested a good candidate to potentiate the efficacy of antimicrobial PSs such as MB [38]. It is worth investigating if it could improve the PDT killing efficacy anaerobically.

Until now, the antibacterial efficacy of most PDTs has been evaluated with single-species planktonic culture or biofilm [27,34,35,36]. Although this type of in vitro model is reproducible in terms of viability and microbial composition [39,40], hence is useful for screening and identifying novel PSs or light sources, the results obtained from this simple model might not be directly translated to the clinical settings because most clinical infections, including periodontitis, are caused by multi-species biofilms [41]. Multi-species biofilms are known to be more resistant to antimicrobial treatments than single-species biofilms. For instance, 12-species biofilms were shown to be considerably less sensitive to PDT killing than single-species biofilms [42]. The saliva-derived multi-species biofilm model used in this study has been shown to allow the development of a complex biofilm containing more than 80 different bacterial species, which could better resemble the multi-species nature of periodontitis [39]. This model has successfully evaluated the antimicrobial efficacy of caries preventive agents [40]. Hence, the findings obtained based on this model are more likely to be implemented in clinic than the commonly used simple model. 

## 5. Conclusions

In conclusion, this study demonstrated that the biofilm killing efficacy of MB-PDT or Cur-PDT was absent under anaerobic conditions. The additional 0.1% HP treatment could (partially) restore the PDT-killing efficacy in a PS-dependent manner. The combined 0.1% HP and PDT treatments may improve the efficacy of PDT for treating patients with aggressive periodontitis or severe cases of chronic periodontitis. 

## Data Availability

The data collected for this study are available from the corresponding author upon request.
